# Double-Stranded Break Repair in Mammalian Cells and Precise Genome Editing

**DOI:** 10.3390/genes13050737

**Published:** 2022-04-22

**Authors:** Akhtar Ali, Wei Xiao, Masroor Ellahi Babar, Yanzhen Bi

**Affiliations:** 1 Key Laboratory of Animal Embryo and Molecular Breeding of Hubei Province, Institute of Animal Science and Veterinary Medicine, Hubei Academy of Agricultural Sciences, Wuhan 430064, China; akhtar.ali@vu.edu.pk (A.A.); fortunatexwei@126.com (W.X.); 2 Department of Biotechnology, Virtual University of Pakistan, Lahore 54000, Pakistan; 3 The University of Agriculture Dera Ismail Khan, Dera Ismail Khan 29220, Pakistan; vc@uad.edu.pk

**Keywords:** DSB repair, NHEJ, HR, RNA template, genome editing

## Abstract

In mammalian cells, double-strand breaks (DSBs) are repaired predominantly by error-prone non-homologous end joining (NHEJ), but less prevalently by error-free template-dependent homologous recombination (HR). DSB repair pathway selection is the bedrock for genome editing. NHEJ results in random mutations when repairing DSB, while HR induces high-fidelity sequence-specific variations, but with an undesirable low efficiency. In this review, we first discuss the latest insights into the action mode of NHEJ and HR in a panoramic view. We then propose the future direction of genome editing by virtue of these advancements. We suggest that by switching NHEJ to HR, full fidelity genome editing and robust gene knock-in could be enabled. We also envision that RNA molecules could be repurposed by RNA-templated DSB repair to mediate precise genetic editing.

## 1. DSB Induction and Pathway Choice

**Figure 1:**
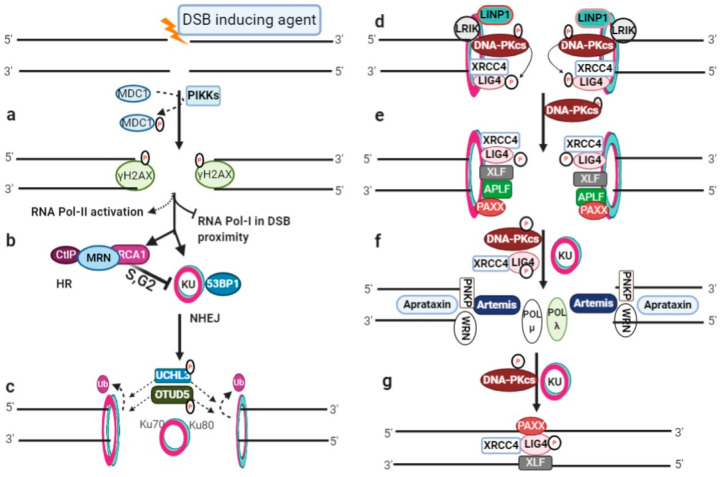
DSB induction and NHEJ repair pathway: (**a**) DSB initiates phosphorylation by activated phophotidylinositol-3 kinase-like kinases (PIKKs) and phosphorylation of H2AX (γH2AX) for cascade signaling; (**b**) RNA polymerase I-based local transcription inhibition in the DSB proximity, RNA polymerase II activation for damage-induced RNA generation, and the recruitment of NHEJ or HR repair machinery; (**c**) KU heterodimer binding at broken ends for NHEJ repair initiation and deubiquitylation of the Ku80 subunit; (**d**) DNA-PKcs autophosphorylates and then phosphorylates LIG4. LINP1 and LRIK are non-coding RNAs that stabilize the KU–DNA complex; (**e**) recruitment of XLF, APLF, and PAXX; (**f**) end processing by Artemis nuclease and other end processing molecules, and gap filling by polymerases; (**g**) LIG4 ligate-processed ends assisted with XRCC4, PAXX and XLF.

In mammalians, genome editing through CRISPR (clustered regularly interspaced short palindromic repeats)-Cas (CRISPR-associated) nuclease systems can induce double-strand breaks (DSBs), initiating one of the conserved repertoire repair pathways, depending on the type of damage, cellular context, and phase of the cell cycle [1,2]. CRISPR/Cas9 genome editing actually takes advantage of DSB repair pathways to introduce variations into the user-defined genomic loci. Eukaryotes possess highly coordinated repair processes, and DSBs can be repaired through non-homologous end joining (NHEJ) or homologous recombination (HR) pathways. Broken DSB ends activate phophotidylinositol-3 kinase-like kinases (PIKKs) (Figure 1a), which phosphorylates histone H2AX (known as γH2AX) leading to cell cycle arrest, a pause in the local transcription of RNA polymerase-I (RNAP-I), RNA polymerase-II (RNAP-II) activation, and up-regulation of genes encoding DNA damage repair factors [3,4,5,6]. γH2AX facilitates in the activation of NHEJ through 53BP1 and KU (Ku70/Ku80) heterodimers [7,8,9], or it can interact with NBS1, through the localization of the MRN (MRE11-RAD50-NBS1) and BRCA1-CtIP (C-terminal binding protein interacting protein) complex at the DSB site to initiate HR repair pathway, as shown in Figure 1b [1].

In this review, we outline the molecular processes of NHEJ and HR and envision the future developments of genome editing by taking advantage of these insightful comprehensions. We also highlight the damage-induced RNAs and RNA-template DSB repair for increasing the genome editing fidelity and efficiency.

## 2. Non-Homologous End Joining (NHEJ)

NHEJ is the predominant, template-independent repair pathway and can potentially re-ligate any type of damaged DNA ends throughout the cell cycle [2,3]. NHEJ can be subdivided into three main sub-sequential steps, namely, (i) DSB end recognition and recruitment of NHEJ machinery, (ii) DNA ends processing, and (iii) DNA ends ligation. The schematic presentation of NHEJ repair pathway is illustrated in Figure 1c–g.

### 2.1. DSB Ends Recognition and Recruitment of NHEJ Machinery

DSB ends are encircled by a stable ring-shaped heterodimer KU protein (150 kDa) composed of Ku70 (70 kDa) and Ku80 (86 kDa) molecules (Figure 1c). Ku70 and Ku80 have a conserved secondary structure; the N-terminal (von Willebrand) and central core bind with DNA while the C-terminal is mainly involved in protein–protein interactions [4,5,6]. KU binding to DSB ends is essential and is an initial key step in NHEJ-repair pathway [7,8,9]. KU binds with the sugar backbone and possesses a high binding affinity with linear dsDNA compared with supercoiled, circular, or ssDNA [4,7,10,11]. KU retention at the broken ends is enhanced by Ku80 deubiquitylation mediated by UCHL3 and OTUD5 [12,13]. This KU–DNA complex acts as scaffold for the recruitment of other NHEJ molecules (Figure 1d). KU recruits DNA-PKcs or XRCC4–LIG4, depending on the nature and/or complexity of the DNA damage [2]. DNA-PKcs autophosphorylates and its retention is facilitated by the Ku80 C-terminal domain [9,14,15,16]. Long noncoding RNAs (LRIK and LINP1) also increase the binding ability of KU with DSB ends [17,18].

The XRCC4–LIG4 complex could also be localized at DSB in a DNA-PKcs-independent manner [19]. The N-terminal domain of XRCC4 interacts with the Ku70 subunit and the C-terminal mediates LIG4 binding with KU [20]. Hence, XRCC4 serves as a tether between KU and LIG4 [8]. LIG4 possesses two BRCT domains known as BRCT1 and BRCT2. The inter-BRCT region and BRCT2 domain bind with the C-terminal region of XRCC4 [21]. The formation of the KU–XRCC4–LIG4 complex is an important rate-limiting step in the DNA-ends joining process. DNA-PKcs phosphorylates LIG4 to stabilize the KU–XRCC4–LIG4 complex [22,23]. 

Other important molecules (Figure 1e) such as XLF and APLF bind with the vWA domain of the Ku80 subunit [24]. In turn, APLF increases XRCC4–LIG4 and XLF retention [25]. XLF interacts with DNA-PKcs [26]. PAXX binds with the Ku70 unit of the KU heterodimer and is stabilized with DNA extension [27]. 

### 2.2. DNA Ends Processing

Depending on the nature of the DNA broken ends, different molecules may be required (Figure 1f) for removing the functional groups involved in blocking ends, resecting ends, making ends ligatable, and gap filling. A number of factors are involved in DNA ends processing (Table 1).

The primary and abundant nuclease that is required is Artemis, which interacts with the FAT domain of DNA-PKcs and the N-terminus region of LIG4 [28,29,30,31]. Mentase and Artemis can trim 3′overhangs, but are more efficient later [32]. Artemis and TDP1 have the ability to catalyze the removal of the 3′phosphoglycolate (3′PG) group from DNA ends [33,34]. Similar to Artemis, the KU–XRCC4–LIG4–APLF complex can also reset 3′overhangs [35]. DNA broken ends may have non-ligatable groups such as 3′phosphate or 5′hydroxyl. These blocking groups can be removed in order to convert into ligatable. PNKP has a kinase domain to incorporate phosphate groups at 5′-OH, preferentially at a double-stranded substrate, while the phosphatase catalytic domain can dephosphorylate single-stranded and double-strand termini 3′-phosphate groups [36]. Aprataxin can specifically release adenylate groups covalently attached with the 5′-phosphate termini of single-strand nicks and gaps to rejoin ends efficiently [37]. The lyase activity of KU has been demonstrated by the Ku80 subunit, which can cleave the apurinic/apyimidinic (AP) site from the partial DNA duplex with 5′- and 3′-protruding ends [38,39]. Gap filling in NHEJ is mainly performed by DNA polymerase µ (Polµ) and λ (Pol λ), which possess functional redundancy [40]. Polµ and Polλ interact with KU and XRCC4–LIG4 via BRCT domains [41,42]. Polλ performs a fill-in activity of terminally compatible overhangs in a template-dependent manner [43]. Polµ has both template-dependent and -independent activities [44,45]. It is also competent to add dNTP as well as rNTP generating regions of the terminal microhomology for subsequent pairing and ligation processes. Thus, Polµ strongly promotes the joining of two DNA strands with incompatible 3′-overhangs [46,47]. TdT also has the ability to incorporate nucleotides in a template-independent manner, but is mainly transcribed in lymphocyte [48].

### 2.3. DNA Ends Ligation

LIG4 is the key molecule and belongs to the superfamily of nucleotidyl transferases that can introduce phosphodiester bonds to join broken ends (Figure 1g). PAXX and XLF are also important components of the ligation process. Despite a small sequence homology, both PAXX and XLF share structural similarity with XRCC4 [49]. PAXX has been demonstrated to promote the ligation of blunt ends in the presence of the KU–XRCC4–LIG4 complex [50]. XLF can stimulate the ligation of incompatible 3′-overhangs interacting with KU and XRCC4 [46,49]. However, in the presence of DNA-PKcs, XLF has the ability to ligate non-cohesive overhangs [51]. Both PAXX and XLF can also promote KU stability and possess redundancy in the ligation of DNA ends [52]. Recently, it has been revealed that XRCC4 is essential for DNA ligation in association with LIG4, PAXX, and XLF [53]. 

**Table 1:** NHEJ-associated key factors with gene location based on human genome assembly (GRCh38.p14) and their functions.

No.	Factor	Gene	Location (*H. sapiens*)	Function in NHEJ
1	DNA-dependent protein kinase catalytic subunit	*PRKDC/XRCC7*	8q11.21	Associated with phosphorylation and essential complex formation [54]
2	Polo-like kinase 1	*PLK1/STPK13*	16p12.2	Phosphorylates DNA-PKcs [55]
3	Polynucleotide kinase/phosphatase	*PNKP/PNK*	19q13.33	Removal of 3′-phosphates [56]
4	Artemis	*DCLRE1C*	10p13	Endonuclease 5′ to 3′ and prevents ends resection [57]
5	DNA ligase IV	*LIG4*	13q33.3	Ligates DNA ends [58]
6	Aprataxin- and PNK-Like Factor	*APLF/PALF*	2p13.3	Scaffold for recruitment of XRCC4–LIG4 and XLF [25] and competent to reset 3′-overhangs [35].
7	Ku70	*XRCC6*	22q13.2	Core component of KU, acts as scaffold for recruitment of NHEJ machinery [59]
8	Ku80	*XRCC5*	2q35	Part of KU and acts as scaffold
9	lncRNA NHEJ pathway 1	*LINP1*	10p14	Modulator scaffold for KU and DNA-PKcs [18,60]
10	Paralog of XRCC4 and XLF	*PAXX/XLS*	9q34.3	Interacts with KU and Polλ to stimulate fill-in gap as well as ends ligation [27,61]
11	Rap1-Interacting Factor 1	*RIF1*	2q23.3	Forms a 53BP1–RIF1 complex to protect from ends resection [62]
12	XRCC4-like factor	*XLF/NHEJ1*	2q35	Interacts with KU and XRCC4 enabling synaptic complex formation required for ends ligation [63,64]
13	X-ray cross complementing protein 4	*XRCC4*	5q14.2	Core component of NHEJ complex [65]
14	Zinc-finger protein 281	*ZNF281/ZBP−99*	1q32.1	Promotes XRCC4 recruitment and interacts with DNA-PKcs and Ku70 [66]
15	Ubiquitin carboxyl terminal hydrolase L3	*UCHL3*	13q22.2	Deubiquitylates Ku80 and enhances its retention [12]
16	OTU Deubiquitinase 5	*OTUD5*	Xp11.23	Deubiquitylates Ku80 and regulates NHEJ [13]
17	TP53 binding protein 1	*53BP1/TP53BP1*	15q15.3	Inhibitor of BRCA1 and key player defining the DSB repair pathway choice [67,68]

## 3. Homologous Recombination (HR)

In eukaryotes, accurate DSB repair is performed by HR, which requires sister-chromatid/repair-template generally restricted to the S/G2 phase of the cell cycle. HR can be categorized into subsections (i) DNA ends resection, (ii) RAD51-nucleofilament formation, (iii) homology searching, (iv) hDNA formation and D-loop extension, and (vi) D-loop dissolution. 

### 3.1. DNA Ends Resection

**Figure 2:**
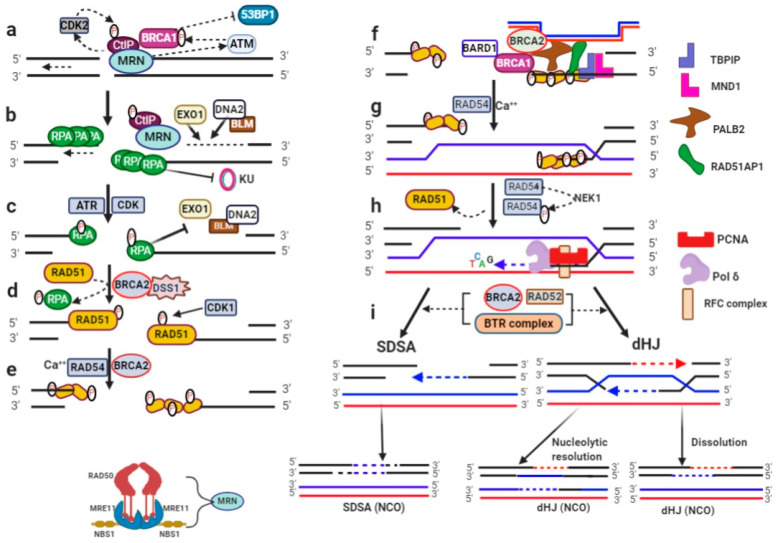
DSB repair via HR: (**a**) MRN complex binding with DSB ends, phosphorylation of CtIP and BRCA1 to activate MRN complex and inhibiting 53BP1; (**b**) long-range resection initiation via the EXO1 or BLM-DNA2 pathways, RPA covering of 3′-ssDNA tails, and KU inhibition; (**c**) RPA phosphorylation (pRPA) by ATR and CDK to negatively regulate resection; (**d**) replacement of pRPA with RAD51 mediated by BRCA2–DSS1 complex; (**e**) RAD51-nucleofilament formation; (**f**) RAD51AP1 and the TBPIP–MND1 complex stabilize RAD51-NF, PALB2 forms a complex with BRCA1, BRCA2, and DNA, and the BRAD1–BRCA1 complex in homology searching; (**g**) synaptic complex and D-loop formation; (**h**) DNA synthesis and D-loop extension; (**i**) D-loop dissociation and annealing via SDSA, in which newly synthesized DNA anneals with ssDNA tails or dHJ formation and dissolution into non-crossover products.

In HR, DSB ends must undergo 5′- and 3′-nucleolytic degradation to generate 3′-ssDNA tails, referred to as ends resection. Activated γH2AX histone interacts with NBS1 and facilitates in the localization of MRN (MRE11–RAD50–NBS1) complex at the DSB site [1]. NBS1 also binds with ATM to render a signaling role of MRN and the recruitment of other molecules [69]. ATM phosphorylates and activates BRCA1 to bind with MRN and facilitates in 53BP1 removal [70], and thus inhibits the NHEJ repair pathway (Figure 2a). RAD50 regulates the MRN affinity with DNA in an ATP-dependent manner [71]. MRE11 binds with DNA ends to perform 3′- and 5′-exonuclease on dsDNA and 5′- and 3′-endonuclease on the ssDNA activity, generating 3′-ssDNA overhangs [72,73]. MRE11 also interacts with CDK2 to facilitate CtIP phosphorylation and stability [74]. This phosphor-mimic CtIP promotes the endonuclease activity of MRN [75]. Short resected 3′-ssDNA tails are covered by RPA in a sequential manner to prevent KU binding, thereby inhibiting the NHEJ repair pathway [76,77].

RPA, MRN, and CtIP stimulate extensive resection preceded by two non-overlapping pathways (Figure 2b). One is executed by EXO1 and the other via DNA2 nuclease in concert with BLM helicase. BLM forms a BTR complex with TOPOIIIα-RMI1/2. BLM enforces 5′- and 3′-resection by DNA2, and also enhances the EXO1 affinity for DNA ends [78]. CtIP influences long-range ends resection beyond MRN regulation, it can activate the BLM helicase and motor activity of DNA2 [79,80]. CtIP also interacts with EXO1 for localization at DSB [81], whereas MRN and RPA stimulate EXO1-dependent resection [78]. CtIP can also inhibit over-resection, thus acting as a DNA resection regulator [82]. 

RPA is necessary for 3′-ssDNA strand protection from degradation, DNA unwinding by BLM, and the accelerated 5′-3′ nucleolytic function of DNA2 [78,83]. However, phosphorylated RPA (pRPA) inhibits BLM and ssDNA accumulation (Figure 2c), therefore pRPA negatively regulates DNA ends resection [84,85].

### 3.2. RAD51-Nucleofilament Formation

After ends resection, the ssDNA–RPA interaction is destabilized and pRPA is replaced with RAD51 [86]. RAD51 mediator proteins stimulate it to form a bridge with ssDNA to displace RPA [87,88]. In vertebrates, BRCA2 is suggested to be a more critical mediator in RPA–RAD51 exchange [89]. It has been revealed that BRCA2 forms a complex with DSS1 (Figure 2d) and replaces RPA with RAD51 [90]. RAD51 forms a nucleoprotein filament (NF), a necessary event for the subsequent homology search, strand invasion, and replication fork protection. RAD51-NF assembly/disassembly represents the ability of rapid and high covering ssDNA via a decreased binding tendency with the DNA sequence. RAD51 ATPases show a complex behavior based on bound nucleotide co-factors during HR [87,91,92]. ATP increases RAD51 affinity with DNA, and RAD51-ATP binds with ssDNA and stretches the DNA B-form length to 150% [93]. RAD51 binds with non-hydrolyzable ATP analogs and forms extended form filaments (99 Å), while the RAD51-ADP filament is compressed and low pitch (76 Å) [92]. RAD51-NF formation is mainly driven by BRCA2, but RAD54 also stabilizes it, independent of its ATPase activity [94,95]. Calcium promotes RAD51-NF formation and stabilizes RAD51-ATP (Figure 2e) [96,97]. 

### 3.3. Homology Searching

RAD51-NF interacts with dsDNA to find a complementary sequence for homology search in the DSB vicinity. In vertebrates, RAD51AP1, TBPIP-MND1 (heterodimer of TBPIP and MND1), and PALB2 (partner and localizer of BRCA2) are important in RAD51-NF bridging with dsDNA (Figure 2f) [98,99,100]. RAD51AP1 is capable of binding with ssDNA and dsDNA, but possesses the highest affinity for branched DNA structures obligatory during HR [101]. TBPIP-MND1 stabilizes the RAD51-NF structure and enhances its ability to capture duplex DNA, which is an essential intermediate step during the synaptic complex [102]. BRCA1 stimulates PALB2 to form a ternary BRCA1–PALB2–BRCA2 complex [103]. PALB2 binds with DNA and stimulates RAD51 recombinase to form a D-loop structure. The BRCA1–BARD1 complex has also been reported in RAD51-mediated homologous pairing [104].

The RAD51–ssDNA–dsDNA complex uses a nucleotide-length specific recognition mechanism for homology searching. The microhomology of the eight-nucleotide tract is sufficient for robust kinetic selection. Successful pairing with the ninth base reduces the binding free energy and the subsequent strand exchange initiates precisely in three base steps, indicating the triplet-base arrangement of the presynaptic complex [105,106]. 

### 3.4. Heteroduplex DNA Formation and D-Loop Extension

During the homology search, RAD51-NF probes and interacts with donor dsDNA to form a synaptic complex. The invading strand 3′-ends intertwine with the donor complementary sequence to form heteroduplex DNA (hDNA). Original base pairing is disrupted during hDNA formation, and this intermediate is known as displacement or D-loop. In humans, RAD51 can accomplish D-loop formation on its own in the presence of Ca++, while RAD54 accelerates the process (Figure 2g) [97,107,108]. RAD54 possesses ATPase to prevent non-productive intermediates during D-loop formation [109], as well as RAD51-promoted translocase activity to create the hDNA junction [110]. In the late G2 phase, never-in-mitosis A-related kinase 1 (NEK1) phosphorylates RAD54 to turn over RAD51 from hDNA to orchestrate HR [108]. 

As shown in Figure 2h, hDNA formation is favored at the 3′-ends sequences compared with the internal homologous region [110]. DNA synthesis essentially requires 3′-ends of invading strands to be intertwined in hDNA, forming a primer–template junction [111]. The minimum required components for D-loop extension, as shown in Figure 2f,g, are RAD51, RAD54, DNA polymerase δ (Polδ), proliferating cell nuclear antigen (PCNA), and replication factor-c (RFC1-5) [112,113,114]. 

Polδ can replicate both invading and complementary strands [115]. Polδ is stimulated and accelerated for hDNA extension by PCNA and its loader RFC1-5 [112]. It was recently revealed that Cft18-RFC and Polϵ form a stable clamp loader/polymerase complex that favors leading strand DNA synthesis, and it is helpful in leveling the PCNA balance in the replication fork [116,117]. Lengths of hDNA sequence vary and may be hundreds of bases long [110]. Extension continues until a topological block is encountered. Topoisomerases can induce negative supercoiling (∼10.5 bp/turn) to relax donor DNA ahead of D-loop extension [118,119]. However, the exact mechanism of bubble migration during DNA synthesis in hDNA is to be determined. Interestingly, MCM8 and MCM9 have been proposed as HR helicases involved downstream of RAD51 processing [120]. However, the detailed mechanism has not been established yet. 

### 3.5. D-Loop Dissolution

D-loop disruption is ultimately required to anneal extended strands with the second resected ends [121,122]. However, it is also essential to remove partial homologous donors and to prevent ssDNA invasion to more than one donor [123]. Additionally, it also decreases the probability that both DSB ends will concurrently invade donor DNA, allowing DNA synthesis as well as maturation to the double Holliday junction (dHJ) and cross-over (CO) outcomes [124,125]. Several proteins are associated with D-loop disruption, but Sgs1, Mph1, and Srs2 are major helicases in *S. cerevisiae* that act in distinct ways, without significant overlapping [126,127,128]. In vertebrates, functional orthologs to Sgs1, Mph1, and Srs2 are BLM, FANCM, and FBH1, respectively [129,130,131]. 

After D-loop disruption, the annealing of nascent DNA homologous is the next step. RAD52 and BRCA2 are the key driving molecules to anneal ssDNA in vertebrates [132,133]. However, second-end annealing is unclear, either by annealing or invasion, regarding restoring the genomic integrity and CO avoidance [118]. It is evident that CO avoidance tendency favors synthesis-dependent strand annealing (SDSA), an alternate processing of extended D-loops by the dissociation and annealing of newly synthesized DNA with ssDNA tails from the other end (Figure 2i) [134]. Evidence shows that all three helicases promote SDSA, thus avoiding a loss of heterozygosity [127]. Although SDSA is a preferred pathway, sometimes dHJs are formed either by second-end annealing with displaced strands of D-loop or by both resected ends to invade donor DNA, and are prolonged through DNA synthesis [135]. These dHJs can be processed to generate CO or non-CO (NCO) products (Figure 2i). The BTR complex has the ability to induce the branch shift of two HJs and to resolve topological limitations resulting in NCO outcomes [136,137]. However, long hDNA sequences favor dHJs and COs [138,139]. These COs may lead to a risk of loss of heterozygosity [140].

## 4. Roles of RNA Transcripts in DSB Repair

### 4.1. dincRNAs in DSB Repair

**Figure 3:**
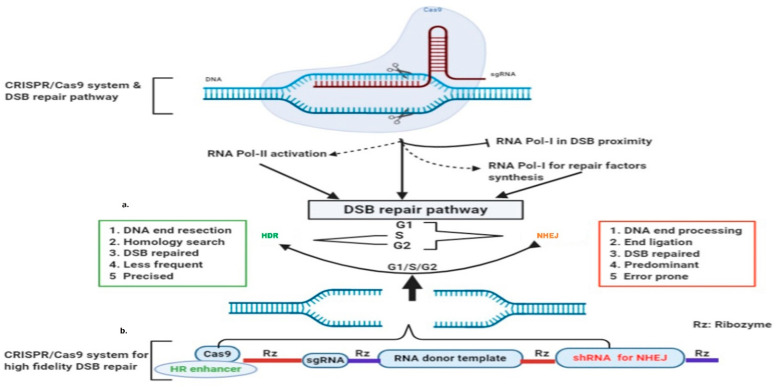
CRISPR-Cas9 induced DSB and RNA-templated high-fidelity repair system: (**a**) Cas9 induced broken ends pause RNA polymerase-I transcription in the DSB vicinity, and activate RNA Polymerase-II and RNA polymerase-I for the synthesis of DSB repair factors; (**b**) single construct for Cas9 expression ribozyme mediated transcription of sgRNA, donor template with desired sequence, and shRNA for NHEJ suppression.

DSB induction inhibits RNA polymerase I (RNAP-I) to down-regulate ongoing local transcription, while it activates RNAP-II promoter-independent activity to generate damage-induced ncRNAs (dincRNA), as shown in Figure 1 and Figure 3 [141]. These dincRNAs can be categorized on the basis of size into long (dilncRNA) >200 nt and small ncRNAs with about 21 nt [142]. Long ncRNAs (lncRNAs) act as a precursor for small ncRNAs, analogous to miRNA biosynthesis [143]. These dincRNAs are exported to the cytoplasm via RNA binding proteins (RBPs), and protect the cell by degrading truncated mRNA [142,144,145]. The role of dincRNAs in the DSB repair process is controversial, as Michelini et al. identified that the MRN complex recruits RNAP-II in the DSB vicinity for DICER or AGO2-mediated HR repair [142]. However, DICER inactivation is reported to reduce 53BP1 foci formation, suggesting its role in the NHEJ repair pathway [146]. Similarly, pre-existing dincRNA transcripts have been reported prior to DSB induction by Bader and Bushell [147], indicating that additional studies are needed in order to understand the detailed molecular mechanisms. 

### 4.2. RNA-Template Mediated DSB Repair

The RNAP-I transcription pause transcribing DSB region is essential to halt truncated mRNA synthesis. Moreover, it is also important, as nascent RNA is proposed to serve as a template [148]. As proposed by Lavigne et al., nascent transcripts or pre-mRNAs are available for transcript-templated repair shortly after the initiating lesion [149]. It is widely accepted that RNAs can pair with homologous DNA strands to form RNA–DNA hybrids [144,150]. RNA-binding proteins (RBPs) such as THRAP3, DICER, DHX9, Drosha, and Senataxin are important driving factors [146,151,152,153,154]. These possess different functions, for example Drosha promotes RNA–DNA hybrids, while Senataxin and RNase H2 resolve [144,154,155,156]. A plethora of studies indicate that RNA–DNA hybrids have direct roles in DSB repair [145,157,158]. Mazina et al. revealed that RPA has a higher RNA binding affinity than DNA, and RAD52 is able to invert the strand exchange between homologous DNA and RNA [159]. It is evident that defective H-RNases could stimulate homologous RNA-mediated DNA repair more than the homologous DNA-template in the presence of RPA [160]. The emerging picture is that dincRNAs are important for truncated mRNA degradation as well as cascade signaling, and RNA molecules homologous to DSB ends could serve as a template for faithful repair through HR. An interesting finding about DNA polymerase θ (Polθ) has revealed its reverse transcription ability, similar to reverse transcriptases in retrovirus. Polθ is efficient in the incorporation of deoxyribonucleotides on RNA versus DNA, and promotes RNA-templated DNA repair in mammalian cells [161]. This suggests the significance of RNA as a template in DNA repair. 

### 4.3. RNA versus DNA as a Donor Repair Template

Both single-stranded oligonucleotides (ssODNs) and double-stranded DNA (dsDNA) are used in genome editing (GE). ssODNs provide higher insertion efficiencies over the dsDNA template [162]. However, ssODNs require efficient delivery in the nuclear region to avoid degradation from ssDNAases [163]. ssODNs can also generate null alleles and are inefficient when applied at scale [164]. The other main disadvantage of ssODNs is their size limitation. Although limited data are available, it has been demonstrated that DSBs can be repaired via the RNA donor template repair pathway [160,165,166].

## 5. DSB-Oriented Genome Editing and Future Prospects

The discovery of the CRISPR/Cas (clustered regularly interspaced short palindromic repeat/CRISPR-associated) system has revolutionized the genome editing (GE), owning to its high efficiency in introducing DSB into the user-defined locus. Accelerated advancements in CRISPR/Cas9 technology have validated its therapeutic applications [167]. Precise GE mainly depends on the DSB repair pathway, and Cas9-induced DSB repair tends to be error prone [168]. Considering the aforementioned DSB repair pathways and the role of RNA molecules, we recommend that faithful and effective GE requires further assistance to ensure HR-mediated DSB repair. Plenty of evidence has revealed that NHEJ is error prone but predominant, so precise editing essentially requires the inhibition of the NHEJ repair pathway. This could be achieved through RNA interference degradation, drug mediated inhibition, or altering the protein sequence of NHEJ driving factors (listed in Table 1) such as 53BP1, DNA-PKcs, Ku70, Ku80, and LIG4 [169,170,171,172,173]. Similarly, plenty of evidence has shown that HR is inherently less efficient in DSB repair, which could be improved through HR enhancers (Table 2). RAD51, RAD52, MRE11, and CtIP have shown a significant increase in template dependent repair when tethered with Cas9 [174,175,176]. Here, we also suggest CtIP as a preferred choice due to its dual role as an HR enhancer and regulator for over-resection [82]. In addition to these, covalently-linked donor repair-oligonucleotides with benzylguanine to Cas9 could also substantially favor HR [177,178]. Single-stranded oligonucleotides (ssODNs) used in GE provide higher insertion efficiencies over the dsDNA template [162]. However, ssODNs require efficient delivery in the nuclear region to avoid degradation from ssDNAases [163]. It has been demonstrated that DSBs can be repaired via the RNA donor template repair pathway [160,165,166]. It is proposed (Figure 3) that nascent RNA or pre-mRNA transcribed from the DSB spanning region could act as a template [149]. In order to enhance co-localization of the RNA donor repair template, we suggest that it could be generated endogenously through ribozymes, and then annealing of this donor to the complement homologous DSB ends can be catalyzed by RAD52 even more efficiently than DNA–DNA [179,180,181,182]. 

We conclude that the silencing of NHEJ driving factors as well as up-regulation of the HR repair pathway through modified Cas9 could be promising to uplift RNA-template-mediated genome editing efficiency and fidelity. 

## Figures and Tables

**Table 2:** Key molecules associated with the HR repair pathway and their gene location based on human genome assembly (GRCh38.p14).

No.	Factor	Gene	Location (*H. sapiens*)	Function in HR
1	Breast cancer gene 1	*BRCA1*	17q21.31	Inhibits 53BP1 to prevent NHEJ
2	Breast cancer gene 2	*BRCA2*	13q13.1	Promotes RPA replacement with RAD51 [90]
3	Bloom’s helicase	*BLM*	15q26.1	Unwinds DNA substrates including Holliday junction [130]
4	CtBP (C-terminal binding protein) interacting protein	*CtIP*	18q11.2	Multifunctional role in HR [79,80]
5	DNA replication helicase/nuclease 2	*DNA2*	10q21.3	Long-range end resection role concerted with BLM [78]
6	Deleted in split hand/split foot type 1	*DSS1*	7q21.3	Forms a complex with BRCA2 to replace RPA with RAD51 [90]
7	Exonuclease 1	*EXO1*	1q43	Executes long-range end resection [183]
8	Meiotic nuclear divisions protein 1	*MND1*	4q31.3	TBPIP-MND1 complex facilitates in homologous strand search [98,102]
9	Meiotic recombination 11	*MRE11*	11q21	Perform nuclease activity generating 3′-ssDNA overhangs [72,73]
10	Nijmegen breakage syndrome 1	*NBS1*	8q21.3	Binds with DSB and ATM to render a signaling role of MRN [69]
11	Partner and localizer of BRCA2	*PALB2*	16p12.2	Forms a BRCA1-PALB2-BRCA2 complex to stimulate RAD51 [103]
12	Proliferating cell nuclear antigen	*PCNA*	20p12.3	Stimulates Polδ-based hDNA extension [112].
13	RAD50	*RAD50*	5q31.1	Forms an ATP-dependent compact structure with dsDNA [71]
14	RAD51	*RAD51*	15q15.1	Forms a nucleoprotein filament (NF), necessary for subsequent homology search, strand invasion, and replication fork protection [87,91,92]
15	RAD51 associated protein 1	*RAD51AP1*	12p13.32	Binds with ssDNA and dsDNA to facilitate D-loop formation [101]
16	RAD54	*RAD54*	Xq21.1	Multifunctional and possess an ATPase activity to prevent non-productive intermediates in D-loop formation [109]
17	Replication protein A1	*RPA1*		Multifunctional, 70kDa main DNA binding subunit of RPA
18	TBP-1 interacting protein	*TBPIP*	17q21.2	TBPIP forms a complex with MND1 during homology search [102]

## Data Availability

Not applicable.

## Abbreviations

DSB	Double-strand break
NHEJ	Non-homologous end joining
HR	Homologous recombination
dsDNA	Double-strand DNA
ssDNA	Single-strand DNA
hDNA	Heteroduplex DNA
KU	Ku70/Ku80 heterodimer
LIG4	DNA ligase 4
MRN	MRE11-RAD50-NBS1
RPA	Replication protein A
dHJ	Double holliday junction
SDSA	Synthesis dependent strand annealing
NCO	Non-crossover
CtIP	CtBP (C-terminal binding protein) interacting protein
UCHL3	Ubiquitin carboxyl-terminal hydrolase isozyme L3
OTUD5	OTU Deubiquitinase 5
DNA-PKcs	DNA-dependent protein kinase catalytic subunit
XRCC4	X-Ray Repair Cross Complementing 4
LIG4	DNA ligase-4
LRIK	lncRNA interacting with Ku
LINP1	LncRNA in non-homologous end joining (NHEJ) pathway 1
BRCT1	BRCA1 C-terminal
BRCT2	BRCA2 C-terminal
XLF	XRCC4-like factor
APLF	Aprataxin and PNKP-like factor
PAXX	Paralog of XRCC4 and XLF
PNKP	Polynucleotide Kinase 3′-Phosphatase
dNTP	Deoxynucleoside triphosphate
rNTP	Ribonucleoside triphosphate
TdT	Terminal deoxynucleotidyl transferase
RAD51	RAD51 Recombinase
MRE11	Meiotic recombination 11
NBS1	Nijmegen breakage syndrome 1 protein
H2AX	H2A histone family member X
CDK2	Cyclin-dependent kinase 2
EXO1	Exonuclease 1
BLM	Bloom Syndrome Helicase
BTR complex	Bloom helicase, topoisomerase IIIα (TOP-IIIα), and the RMI1/2
TOPOIIIα	Topoisomerase IIIα
BRCA1	Breast Cancer 1
BRCA2	Breast Cancer 2
RAD51-NF	RAD51-nucleoprotein filament
RAD51AP1	RAD51 associated protein 1
TBPIP	TBP-1 interacting protein
MND1	Meiotic nuclear divisions protein 1
PALB2	Partner and Localizer of BRCA2
NEK1	Never-in-mitosis A-related kinase 1
POL δ	DNA polymerase δ
PCNA	Proliferating cell nuclear antigen
RFC1-5	Replication factor-c 1-5
dilncRNA	Damage-induced long ncRNAs
DICER	Endoribonuclease encoded by DICER1 gene
AGO2	Argonaute RISC catalytic component 2
THRAP3	Thyroid hormone receptor associated protein 3
DHX9	DExH-Box Helicase 9
ssODNs	Single-stranded oligonucleotides
FANCM	Fanconi anemia, complementation group M
FBH1	F-box DNA helicase 1

## References

[1] Kobayashi J., Tauchi H., Sakamoto S., Nakamura A., Morishima K., Matsuura S., Kobayashi T., Tamai K., Tanimoto K., Komatsu K. (2002). NBS1 localizes to gamma-H2AX foci through interaction with the FHA/BRCT domain. Curr. Biol..

[2] Davis A.J., Chen D.J. (2013). DNA double strand break repair via non-homologous end-joining. Transl. Cancer Res..

[3] Reid D.A., Keegan S., Leo-Macias A., Watanabe G., Strande N.T., Chang H.H., Oksuz B.A., Fenyo D., Lieber M.R., Ramsden D.A. (2015). Organization and dynamics of the nonhomologous end-joining machinery during DNA double-strand break repair. Proc. Natl. Acad. Sci. USA.

[4] Downs J.A., Jackson S.P. (2004). A means to a DNA end: The many roles of Ku. Nat. Rev. Mol. Cell Biol..

[5] Fell V.L., Schild-Poulter C. (2015). The Ku heterodimer: Function in DNA repair and beyond. Mutat. Res. Rev. Mutat. Res..

[6] Zhang Z., Hu W., Cano L., Lee T.D., Chen D.J., Chen Y. (2004). Solution structure of the C-terminal domain of Ku80 suggests important sites for protein-protein interactions. Structure.

[7] Ono M., Tucker P.W., Capra J.D. (1994). Production and characterization of recombinant human Ku antigen. Nucleic Acids Res..

[8] Mari P.-O., Florea B.I., Persengiev S.P., Verkaik N.S., Brüggenwirth H.T., Modesti M., Giglia-Mari G., Bezstarosti K., Demmers J.A.A., Luider T.M. (2006). Dynamic assembly of end-joining complexes requires interaction between Ku70/80 and XRCC4. Proc. Natl. Acad. Sci. USA.

[9] Uematsu N., Weterings E., Yano K., Morotomi-Yano K., Jakob B., Taucher-Scholz G., Mari P.O., van Gent D.C., Chen B.P., Chen D.J. (2007). Autophosphorylation of DNA-PKCS regulates its dynamics at DNA double-strand breaks. J. Cell Biol..

[10] Walker J.R., Corpina R.A., Goldberg J. (2001). Structure of the Ku heterodimer bound to DNA and its implications for double-strand break repair. Nature.

[11] Blier P.R., Griffith A.J., Craft J., Hardin J.A. (1993). Binding of Ku protein to DNA. Measurement of affinity for ends and demonstration of binding to nicks. J. Biol. Chem..

[12] Nishi R., Wijnhoven P.W.G., Kimura Y., Matsui M., Konietzny R., Wu Q., Nakamura K., Blundell T.L., Kessler B.M. (2018). The deubiquitylating enzyme UCHL3 regulates Ku80 retention at sites of DNA damage. Sci. Rep..

[13] Li F., Sun Q., Liu K., Han H., Lin N., Cheng Z., Cai Y., Tian F., Mao Z., Tong T. (2019). The deubiquitinase OTUD5 regulates Ku80 stability and non-homologous end joining. Cell. Mol. Life Sci..

[14] Weterings E., van Gent D.C. (2004). The mechanism of non-homologous end-joining: A synopsis of synapsis. DNA Repair.

[15] Hammel M., Yu Y., Mahaney B.L., Cai B., Ye R., Phipps B.M., Rambo R.P., Hura G.L., Pelikan M., So S. (2010). Ku and DNA-dependent protein kinase dynamic conformations and assembly regulate DNA binding and the initial non-homologous end joining complex. J. Biol. Chem..

[16] Shao Z., Flynn R.A., Crowe J.L., Zhu Y., Liang J., Jiang W., Aryan F., Aoude P., Bertozzi C.R., Estes V.M. (2020). DNA-PKcs has KU-dependent function in rRNA processing and haematopoiesis. Nature.

[17] Wang D., Zhou Z., Wu E., Ouyang C., Wei G., Wang Y., He D., Cui Y., Zhang D., Chen X. (2020). LRIK interacts with the Ku70–Ku80 heterodimer enhancing the efficiency of NHEJ repair. Cell Death Differ..

[18] Zhang Y., He Q., Hu Z., Feng Y., Fan L., Tang Z., Yuan J., Shan W., Li C., Hu X. (2016). Long noncoding RNA LINP1 regulates repair of DNA double-strand breaks in triple-negative breast cancer. Nat. Struct. Mol. Biol..

[19] Yano K., Chen D.J. (2008). Live cell imaging of XLF and XRCC4 reveals a novel view of protein assembly in the non-homologous end-joining pathway. Cell Cycle.

[20] Junop M.S., Modesti M., Guarné A., Ghirlando R., Gellert M., Yang W. (2000). Crystal structure of the Xrcc4 DNA repair protein and implications for end joining. EMBO J..

[21] Sibanda B.L., Critchlow S.E., Begun J., Pei X.Y., Jackson S.P., Blundell T.L., Pellegrini L. (2001). Crystal structure of an Xrcc4-DNA ligase IV complex. Nat. Struct. Biol..

[22] Nick McElhinny S.A., Snowden C.M., McCarville J., Ramsden D.A. (2000). Ku recruits the XRCC4-ligase IV complex to DNA ends. Mol. Cell. Biol..

[23] Costantini S., Woodbine L., Andreoli L., Jeggo P.A., Vindigni A. (2007). Interaction of the Ku heterodimer with the DNA ligase IV/Xrcc4 complex and its regulation by DNA-PK. DNA Repair.

[24] Nemoz C., Ropars V., Frit P., Gontier A., Drevet P., Yu J., Guerois R., Pitois A., Comte A., Delteil C. (2018). XLF and APLF bind Ku80 at two remote sites to ensure DNA repair by non-homologous end joining. Nat. Struct. Mol. Biol..

[25] Grundy G.J., Rulten S.L., Zeng Z., Arribas-Bosacoma R., Iles N., Manley K., Oliver A., Caldecott K.W. (2013). APLF promotes the assembly and activity of non-homologous end joining protein complexes. EMBO J..

[26] Xing M., Oksenych V. (2019). Genetic interaction between DNA repair factors PAXX, XLF, XRCC4 and DNA-PKcs in human cells. FEBS Open Bio..

[27] Tadi S.K., Tellier-Lebègue C., Nemoz C., Drevet P., Audebert S., Roy S., Meek K., Charbonnier J.-B., Modesti M. (2016). PAXX Is an Accessory c-NHEJ Factor that Associates with Ku70 and Has Overlapping Functions with XLF. Cell Rep..

[28] Chang H.H.Y., Lieber M.R. (2016). Structure-Specific nuclease activities of Artemis and the Artemis: DNA-PKcs complex. Nucleic Acids Res..

[29] Chang H.H.Y., Watanabe G., Lieber M.R. (2015). Unifying the DNA end-processing roles of the artemis nuclease: Ku-dependent artemis resection at blunt DNA ends. J. Biol. Chem..

[30] Dominski Z. (2007). Nucleases of the Metallo-β-lactamase Family and Their Role in DNA and RNA Metabolism. Crit. Rev. Biochem. Mol. Biol..

[31] De Ioannes P., Malu S., Cortes P., Aggarwal A.K. (2012). Structural basis of DNA ligase IV-Artemis interaction in nonhomologous end-joining. Cell Rep..

[32] Mohapatra S., Yannone S.M., Lee S.H., Hromas R.A., Akopiants K., Menon V., Ramsden D.A., Povirk L.F. (2013). Trimming of damaged 3′ overhangs of DNA double-strand breaks by the Metnase and Artemis endonucleases. DNA Repair.

[33] Povirk L.F., Zhou T., Zhou R., Cowan M.J., Yannone S.M. (2007). Processing of 3′-phosphoglycolate-terminated DNA double strand breaks by Artemis nuclease. J. Biol. Chem..

[34] Inamdar K.V., Pouliot J.J., Zhou T., Lees-Miller S.P., Rasouli-Nia A., Povirk L.F. (2002). Conversion of phosphoglycolate to phosphate termini on 3′ overhangs of DNA double strand breaks by the human tyrosyl-DNA phosphodiesterase hTdp1. J. Biol. Chem..

[35] Li S., Kanno S., Watanabe R., Ogiwara H., Kohno T., Watanabe G., Yasui A., Lieber M.R. (2011). Polynucleotide kinase and aprataxin-like forkhead-associated protein (PALF) acts as both a single-stranded DNA endonuclease and a single-stranded DNA 3′ exonuclease and can participate in DNA end joining in a biochemical system. J. Biol. Chem..

[36] Bernstein N.K., Williams R.S., Rakovszky M.L., Cui D., Green R., Karimi-Busheri F., Mani R.S., Galicia S., Koch C.A., Cass C.E. (2005). The molecular architecture of the mammalian DNA repair enzyme, polynucleotide kinase. Mol. Cell.

[37] Ahel I., Rass U., El-Khamisy S.F., Katyal S., Clements P.M., McKinnon P.J., Caldecott K.W., West S.C. (2006). The neurodegenerative disease protein aprataxin resolves abortive DNA ligation intermediates. Nature.

[38] Kosova A.A., Khodyreva S.N., Lavrik O.I. (2016). Ku antigen displays the AP lyase activity on a certain type of duplex DNA. Biochim. Biophys. Acta.

[39] Roberts S.A., Strande N., Burkhalter M.D., Strom C., Havener J.M., Hasty P., Ramsden D.A. (2010). Ku is a 5′-dRP/AP lyase that excises nucleotide damage near broken ends. Nature.

[40] Bebenek K., Pedersen L.C., Kunkel T.A. (2014). Structure-function studies of DNA polymerase λ. Biochemistry.

[41] Nick McElhinny S.A., Havener J.M., Garcia-Diaz M., Juárez R., Bebenek K., Kee B.L., Blanco L., Kunkel T.A., Ramsden D.A. (2005). A gradient of template dependence defines distinct biological roles for family X polymerases in nonhomologous end joining. Mol. Cell.

[42] Fan W., Wu X. (2004). DNA polymerase lambda can elongate on DNA substrates mimicking non-homologous end joining and interact with XRCC4-ligase IV complex. Biochem. Biophys. Res. Commun..

[43] García-Díaz M., Bebenek K., Sabariegos R., Domínguez O., Rodríguez J., Kirchhoff T., García-Palomero E., Picher A.J., Juárez R., Ruiz J.F. (2002). DNA polymerase lambda, a novel DNA repair enzyme in human cells. J. Biol. Chem..

[44] Domínguez O., Ruiz J.F., Laín de Lera T., García-Díaz M., González M.A., Kirchhoff T., Martínez-A C., Bernad A., Blanco L. (2000). DNA polymerase mu (Pol mu), homologous to TdT, could act as a DNA mutator in eukaryotic cells. EMBO J..

[45] Mahajan K.N., Nick McElhinny S.A., Mitchell B.S., Ramsden D.A. (2002). Association of DNA polymerase mu (pol mu) with Ku and ligase IV: Role for pol mu in end-joining double-strand break repair. Mol. Cell. Biol..

[46] Gu J., Lu H., Tippin B., Shimazaki N., Goodman M.F., Lieber M.R. (2007). XRCC4:DNA ligase IV can ligate incompatible DNA ends and can ligate across gaps. EMBO J..

[47] Pryor J.M., Waters C.A., Aza A., Asagoshi K., Strom C., Mieczkowski P.A., Blanco L., Ramsden D.A. (2015). Essential role for polymerase specialization in cellular nonhomologous end joining. Proc. Natl. Acad. Sci. USA.

[48] Nourrit F., Coquilleau I., D’Andon M.F., Rougeon F., Doyen N. (1999). Methylation of the promoter region may be involved in tissue-specific expression of the mouse terminal deoxynucleotidyl transferase gene. J. Mol. Biol..

[49] Andres S.N., Modesti M., Tsai C.J., Chu G., Junop M.S. (2007). Crystal structure of human XLF: A twist in nonhomologous DNA end-joining. Mol. Cell.

[50] Ochi T., Blackford A.N., Coates J., Jhujh S., Mehmood S., Tamura N., Travers J., Wu Q., Draviam V.M., Robinson C.V. (2015). DNA repair. PAXX, a paralog of XRCC4 and XLF, interacts with Ku to promote DNA double-strand break repair. Science.

[51] Tsai C.J., Kim S.A., Chu G. (2007). Cernunnos/XLF promotes the ligation of mismatched and noncohesive DNA ends. Proc. Natl. Acad. Sci. USA.

[52] Hung P.J., Chen B.R., George R., Liberman C., Morales A.J., Colon-Ortiz P., Tyler J.K., Sleckman B.P., Bredemeyer A.L. (2017). Deficiency of XLF and PAXX prevents DNA double-strand break repair by non-homologous end joining in lymphocytes. Cell Cycle.

[53] Ruis B., Molan A., Takasugi T., Hendrickson E.A. (2020). Absence of XRCC4 and its paralogs in human cells reveal differences in outcomes for DNA repair and V(D)J recombination. DNA Repair.

[54] Davis A.J., Chen B.P.C., Chen D.J. (2014). DNA-PK: A dynamic enzyme in a versatile DSB repair pathway. DNA Repair.

[55] Douglas P., Ye R., Trinkle-Mulcahy L., Neal J.A., De Wever V., Morrice N.A., Meek K., Lees-Miller S.P. (2014). Polo-like kinase 1 (PLK1) and protein phosphatase 6 (PP6) regulate DNA-dependent protein kinase catalytic subunit (DNA-PKcs) phosphorylation in mitosis. Biosci. Rep..

[56] Li M., Lu L.Y., Yang C.Y., Wang S., Yu X. (2013). The FHA and BRCT domains recognize ADP-ribosylation during DNA damage response. Genes Dev..

[57] Wang J., Aroumougame A., Lobrich M., Li Y., Chen D., Chen J., Gong Z. (2014). PTIP associates with Artemis to dictate DNA repair pathway choice. Genes Dev..

[58] Altmann T., Gennery A.R. (2016). DNA ligase IV syndrome; a review. Orphanet J. Rare Dis..

[59] Wang C., Lees-Miller S.P. (2013). Detection and repair of ionizing radiation-induced DNA double strand breaks: New developments in nonhomologous end joining. Int. J. Radiat. Oncol. Biol. Phys..

[60] Sakthianandeswaren A., Liu S., Sieber O.M. (2016). Long noncoding RNA LINP1: Scaffolding non-homologous end joining. Cell Death Discov..

[61] Craxton A., Munnur D., Jukes-Jones R., Skalka G., Langlais C., Cain K., Malewicz M. (2018). PAXX and its paralogs synergistically direct DNA polymerase λ activity in DNA repair. Nat. Commun..

[62] Chapman J.R., Barral P., Vannier J.B., Borel V., Steger M., Tomas-Loba A., Sartori A.A., Adams I.R., Batista F.D., Boulton S.J. (2013). RIF1 is essential for 53BP1-dependent nonhomologous end joining and suppression of DNA double-strand break resection. Mol. Cell.

[63] Yang S., Wang X.Q. (2017). XLF-mediated NHEJ activity in hepatocellular carcinoma therapy resistance. BMC Cancer.

[64] Carney S.M., Moreno A.T., Piatt S.C., Cisneros-Aguirre M., Lopezcolorado F.W., Stark J.M., Loparo J.J. (2020). XLF acts as a flexible connector during non-homologous end joining. bioRxiv.

[65] Soulas-Sprauel P., Le Guyader G., Rivera-Munoz P., Abramowski V., Olivier-Martin C., Goujet-Zalc C., Charneau P., de Villartay J.-P. (2007). Role for DNA repair factor XRCC4 in immunoglobulin class switch recombination. J. Exp. Med..

[66] Nicolai S., Mahen R., Raschellà G., Marini A., Pieraccioli M., Malewicz M., Venkitaraman A.R., Melino G. (2020). ZNF281 is recruited on DNA breaks to facilitate DNA repair by non-homologous end joining. Oncogene.

[67] Wang B., Matsuoka S., Carpenter P.B., Elledge S.J. (2002). 53BP1, a Mediator of the DNA Damage Checkpoint. Science.

[68] Gupta A., Hunt C.R., Chakraborty S., Pandita R.K., Yordy J., Ramnarain D.B., Horikoshi N., Pandita T.K. (2014). Role of 53BP1 in the regulation of DNA double-strand break repair pathway choice. Radiat. Res..

[69] Lee J.H., Paull T.T. (2004). Direct activation of the ATM protein kinase by the Mre11/Rad50/Nbs1 complex. Science.

[70] Chappell W.H., Gautam D., Ok S.T., Johnson B.A., Anacker D.C., Moody C.A. (2015). Homologous Recombination Repair Factors Rad51 and BRCA1 Are Necessary for Productive Replication of Human Papillomavirus 31. J. Virol..

[71] Shibata A., Moiani D., Arvai A.S., Perry J., Harding S.M., Genois M.-M., Maity R., van Rossum-Fikkert S., Kertokalio A., Romoli F. (2014). DNA double-strand break repair pathway choice is directed by distinct MRE11 nuclease activities. Mol. Cell.

[72] Furuse M., Nagase Y., Tsubouchi H., Murakami-Murofushi K., Shibata T., Ohta K. (1998). Distinct roles of two separable in vitro activities of yeast Mre11 in mitotic and meiotic recombination. EMBO J..

[73] Mirzoeva O.K., Petrini J.H. (2001). DNA damage-dependent nuclear dynamics of the Mre11 complex. Mol. Cell. Biol..

[74] Buis J., Stoneham T., Spehalski E., Ferguson D.O. (2012). Mre11 regulates CtIP-dependent double-strand break repair by interaction with CDK2. Nat. Struct. Mol. Biol..

[75] Anand R., Ranjha L., Cannavo E., Cejka P. (2016). Phosphorylated CtIP Functions as a Co-factor of the MRE11-RAD50-NBS1 Endonuclease in DNA End Resection. Mol. Cell.

[76] Krasner D.S., Daley J.M., Sung P., Niu H. (2015). Interplay between Ku and Replication Protein A in the Restriction of Exo1-mediated DNA Break End Resection. J. Biol. Chem..

[77] Bochkarev A., Pfuetzner R.A., Edwards A.M., Frappier L. (1997). Structure of the single-stranded-DNA-binding domain of replication protein A bound to DNA. Nature.

[78] Nimonkar A.V., Genschel J., Kinoshita E., Polaczek P., Campbell J.L., Wyman C., Modrich P., Kowalczykowski S.C. (2011). BLM-DNA2-RPA-MRN and EXO1-BLM-RPA-MRN constitute two DNA end resection machineries for human DNA break repair. Genes Dev..

[79] Daley J.M., Jimenez-Sainz J., Wang W., Miller A.S., Xue X., Nguyen K.A., Jensen R.B., Sung P. (2017). Enhancement of BLM-DNA2-Mediated Long-Range DNA End Resection by CtIP. Cell Rep..

[80] Ceppi I., Howard S.M., Kasaciunaite K., Pinto C., Anand R., Seidel R., Cejka P. (2020). CtIP promotes the motor activity of DNA2 to accelerate long-range DNA end resection. Proc. Natl. Acad. Sci. USA.

[81] Eid W., Steger M., El-Shemerly M., Ferretti L.P., Peña-Diaz J., König C., Valtorta E., Sartori A.A., Ferrari S. (2010). DNA end resection by CtIP and exonuclease 1 prevents genomic instability. EMBO Rep..

[82] Howard S.M., Ceppi I., Anand R., Geiger R., Cejka P. (2020). The internal region of CtIP negatively regulates DNA end resection. Nucleic Acids Res..

[83] Chen H., Lisby M., Symington L.S. (2013). RPA coordinates DNA end resection and prevents formation of DNA hairpins. Mol. Cell.

[84] Soniat M.M., Myler L.R., Kuo H.-C., Paull T.T., Finkelstein I.J. (2019). RPA Phosphorylation Inhibits DNA Resection. Mol. Cell.

[85] Vassin V.M., Anantha R.W., Sokolova E., Kanner S., Borowiec J.A. (2009). Human RPA phosphorylation by ATR stimulates DNA synthesis and prevents ssDNA accumulation during DNA-replication stress. J. Cell Sci..

[86] Bell J.C., Kowalczykowski S.C. (2016). Mechanics and Single-Molecule Interrogation of DNA Recombination. Annu. Rev. Biochem..

[87] Sung P., Krejci L., Van Komen S., Sehorn M.G. (2003). Rad51 recombinase and recombination mediators. J. Biol. Chem..

[88] Liu J., Ehmsen K.T., Heyer W.D., Morrical S.W. (2011). Presynaptic filament dynamics in homologous recombination and DNA repair. Crit. Rev. Biochem. Mol. Biol..

[89] Jensen R.B., Ozes A., Kim T., Estep A., Kowalczykowski S.C. (2013). BRCA2 is epistatic to the RAD51 paralogs in response to DNA damage. DNA Repair.

[90] Zhao W., Vaithiyalingam S., San Filippo J., Maranon D.G., Jimenez-Sainz J., Fontenay G.V., Kwon Y., Leung S.G., Lu L., Jensen R.B. (2015). Promotion of BRCA2-Dependent Homologous Recombination by DSS1 via RPA Targeting and DNA Mimicry. Mol. Cell.

[91] Špírek M., Mlcoušková J., Belán O., Gyimesi M., Harami G.M., Molnár E., Novacek J., Kovács M., Krejci L. (2018). Human RAD51 rapidly forms intrinsically dynamic nucleoprotein filaments modulated by nucleotide binding state. Nucleic Acids Res..

[92] Yu X., Jacobs S.A., West S.C., Ogawa T., Egelman E.H. (2001). Domain structure and dynamics in the helical filaments formed by RecA and Rad51 on DNA. Proc. Natl. Acad. Sci. USA.

[93] Chen Z., Yang H., Pavletich N.P. (2008). Mechanism of homologous recombination from the RecA-ssDNA/dsDNA structures. Nature.

[94] Mazin A.V., Alexeev A.A., Kowalczykowski S.C. (2003). A novel function of Rad54 protein. Stabilization of the Rad51 nucleoprotein filament. J. Biol. Chem..

[95] Sanchez H., Kertokalio A., van Rossum-Fikkert S., Kanaar R., Wyman C. (2013). Combined optical and topographic imaging reveals different arrangements of human RAD54 with presynaptic and postsynaptic RAD51-DNA filaments. Proc. Natl. Acad. Sci. USA.

[96] Ristic D., Modesti M., van der Heijden T., van Noort J., Dekker C., Kanaar R., Wyman C. (2005). Human Rad51 filaments on double- and single-stranded DNA: Correlating regular and irregular forms with recombination function. Nucleic Acids Res..

[97] Bugreev D.V., Mazin A.V. (2004). Ca^2+^ activates human homologous recombination protein Rad51 by modulating its ATPase activity. Proc. Natl. Acad. Sci. USA.

[98] Chi P., San Filippo J., Sehorn M.G., Petukhova G.V., Sung P. (2007). Bipartite stimulatory action of the Hop2-Mnd1 complex on the Rad51 recombinase. Genes Dev..

[99] Pires E., Sung P., Wiese C. (2017). Role of RAD51AP1 in homologous recombination DNA repair and carcinogenesis. DNA Repair.

[100] Sy S.M.H., Huen M.S.Y., Chen J. (2009). PALB2 is an integral component of the BRCA complex required for homologous recombination repair. Proc. Natl. Acad. Sci. USA.

[101] Modesti M., Budzowska M., Baldeyron C., Demmers J.A.A., Ghirlando R., Kanaar R. (2007). RAD51AP1 Is a Structure-Specific DNA Binding Protein that Stimulates Joint Molecule Formation during RAD51-Mediated Homologous Recombination. Mol. Cell.

[102] Enomoto R., Kinebuchi T., Sato M., Yagi H., Kurumizaka H., Yokoyama S. (2006). Stimulation of DNA Strand Exchange by the Human TBPIP/Hop2-Mnd1 Complex. J. Biol. Chem..

[103] Zhang F., Ma J., Wu J., Ye L., Cai H., Xia B., Yu X. (2009). PALB2 links BRCA1 and BRCA2 in the DNA-damage response. Curr. Biol. CB.

[104] Zhao W., Steinfeld J.B., Liang F., Chen X., Maranon D.G., Jian Ma C., Kwon Y., Rao T., Wang W., Sheng C. (2017). BRCA1-BARD1 promotes RAD51-mediated homologous DNA pairing. Nature.

[105] Qi Z., Redding S., Lee J.Y., Gibb B., Kwon Y., Niu H., Gaines W.A., Sung P., Greene E.C. (2015). DNA sequence alignment by microhomology sampling during homologous recombination. Cell.

[106] Greene E.C. (2016). DNA Sequence Alignment during Homologous Recombination. J. Biol. Chem..

[107] Mazina O.M., Mazin A.V. (2004). Human Rad54 Protein Stimulates DNA Strand Exchange Activity of hRad51 Protein in the Presence of Ca^2+^. J. Biol. Chem..

[108] Spies J., Waizenegger A., Barton O., Sürder M., Wright W.D., Heyer W.-D., Löbrich M. (2016). Nek1 Regulates Rad54 to Orchestrate Homologous Recombination and Replication Fork Stability. Mol. Cell.

[109] Tavares E.M., Wright W.D., Heyer W.-D., Le Cam E., Dupaigne P. (2019). In vitro role of Rad54 in Rad51-ssDNA filament-dependent homology search and synaptic complexes formation. Nat. Commun..

[110] Wright W.D., Heyer W.D. (2014). Rad54 functions as a heteroduplex DNA pump modulated by its DNA substrates and Rad51 during D loop formation. Mol. Cell.

[111] McVey M., Khodaverdian V.Y., Meyer D., Cerqueira P.G., Heyer W.D. (2016). Eukaryotic DNA Polymerases in Homologous Recombination. Annu. Rev. Genet..

[112] Li X., Stith C.M., Burgers P.M., Heyer W.D. (2009). PCNA is required for initiation of recombination-associated DNA synthesis by DNA polymerase delta. Mol. Cell.

[113] Sneeden J.L., Grossi S.M., Tappin I., Hurwitz J., Heyer W.D. (2013). Reconstitution of recombination-associated DNA synthesis with human proteins. Nucleic Acids Res..

[114] Sebesta M., Burkovics P., Haracska L., Krejci L. (2011). Reconstitution of DNA repair synthesis in vitro and the role of polymerase and helicase activities. DNA Repair.

[115] Donnianni R.A., Zhou Z.X., Lujan S.A., Al-Zain A., Garcia V., Glancy E., Burkholder A.B., Kunkel T.A., Symington L.S. (2019). DNA Polymerase Delta Synthesizes Both Strands during Break-Induced Replication. Mol. Cell.

[116] Stokes K., Winczura A., Song B., Piccoli G.D., Grabarczyk D.B. (2020). Ctf18-RFC and DNA Pol ϵ form a stable leading strand polymerase/clamp loader complex required for normal and perturbed DNA replication. Nucleic Acids Res..

[117] Liu H.W., Bouchoux C., Panarotto M., Kakui Y., Patel H., Uhlmann F. (2020). Division of Labor between PCNA Loaders in DNA Replication and Sister Chromatid Cohesion Establishment. Mol. Cell.

[118] Wright W.D., Shah S.S., Heyer W.-D. (2018). Homologous recombination and the repair of DNA double-strand breaks. J. Biol. Chem..

[119] Fasching C.L., Cejka P., Kowalczykowski S.C., Heyer W.D. (2015). Top3-Rmi1 dissolve Rad51-mediated D loops by a topoisomerase-based mechanism. Mol. Cell.

[120] Natsume T., Nishimura K., Minocherhomji S., Bhowmick R., Hickson I.D., Kanemaki M.T. (2017). Acute inactivation of the replicative helicase in human cells triggers MCM8-9-dependent DNA synthesis. Genes Dev..

[121] McVey M., Adams M., Staeva-Vieira E., Sekelsky J.J. (2004). Evidence for multiple cycles of strand invasion during repair of double-strand gaps in Drosophila. Genetics.

[122] Smith C.E., Llorente B., Symington L.S. (2007). Template switching during break-induced replication. Nature.

[123] Piazza A., Wright W.D., Heyer W.D. (2017). Multi-invasions Are Recombination Byproducts that Induce Chromosomal Rearrangements. Cell.

[124] Pâques F., Haber J.E. (1999). Multiple pathways of recombination induced by double-strand breaks in Saccharomyces cerevisiae. Microbiol. Mol. Biol. Rev..

[125] Szostak J.W., Orr-Weaver T.L., Rothstein R.J., Stahl F.W. (1983). The double-strand-break repair model for recombination. Cell.

[126] Ira G., Malkova A., Liberi G., Foiani M., Haber J.E. (2003). Srs2 and Sgs1-Top3 suppress crossovers during double-strand break repair in yeast. Cell.

[127] Mitchel K., Lehner K., Jinks-Robertson S. (2013). Heteroduplex DNA position defines the roles of the Sgs1, Srs2, and Mph1 helicases in promoting distinct recombination outcomes. PLoS Genet..

[128] Prakash R., Satory D., Dray E., Papusha A., Scheller J., Kramer W., Krejci L., Klein H., Haber J.E., Sung P. (2009). Yeast Mph1 helicase dissociates Rad51-made D-loops: Implications for crossover control in mitotic recombination. Genes Dev..

[129] Chiolo I., Saponaro M., Baryshnikova A., Kim J.-H., Seo Y.-S., Liberi G. (2007). The human F-Box DNA helicase FBH1 faces Saccharomyces cerevisiae Srs2 and postreplication repair pathway roles. Mol. Cell. Biol..

[130] Watt P.M., Hickson I.D., Borts R.H., Louis E.J. (1996). SGS1, a homologue of the Bloom’s and Werner’s syndrome genes, is required for maintenance of genome stability in Saccharomyces cerevisiae. Genetics.

[131] Deans A.J., West S.C. (2009). FANCM Connects the Genome Instability Disorders Bloom’s Syndrome and Fanconi Anemia. Mol. Cell.

[132] Rothenberg E., Grimme J.M., Spies M., Ha T. (2008). Human Rad52-mediated homology search and annealing occurs by continuous interactions between overlapping nucleoprotein complexes. Proc. Natl. Acad. Sci. USA.

[133] Jensen R.B., Carreira A., Kowalczykowski S.C. (2010). Purified human BRCA2 stimulates RAD51-mediated recombination. Nature.

[134] Jenkins S.S., Mukherjee S., Heyer W.D., Bradshaw R.A., Stahl P.D. (2016). DNA Repair by Homologous Recombination. Encyclopedia of Cell Biology.

[135] Bzymek M., Thayer N.H., Oh S.D., Kleckner N., Hunter N. (2010). Double Holliday junctions are intermediates of DNA break repair. Nature.

[136] Bizard A.H., Hickson I.D. (2014). The dissolution of double Holliday junctions. Cold Spring Harb. Perspect. Biol..

[137] Wu L., Hickson I.D. (2003). The Bloom’s syndrome helicase suppresses crossing over during homologous recombination. Nature.

[138] Neuwirth E.A., Honma M., Grosovsky A.J. (2007). Interchromosomal crossover in human cells is associated with long gene conversion tracts. Mol. Cell. Biol..

[139] Aguilera A., Klein H.L. (1989). Yeast intrachromosomal recombination: Long gene conversion tracts are preferentially associated with reciprocal exchange and require the RAD1 and RAD3 gene products. Genetics.

[140] Moynahan M.E., Jasin M. (2010). Mitotic homologous recombination maintains genomic stability and suppresses tumorigenesis. Nat. Rev. Mol. Cell Biol..

[141] Shanbhag N.M., Rafalska-Metcalf I.U., Balane-Bolivar C., Janicki S.M., Greenberg R.A. (2010). ATM-Dependent Chromatin Changes Silence Transcription In cis to DNA Double-Strand Breaks. Cell.

[142] Michelini F., Pitchiaya S., Vitelli V., Sharma S., Gioia U., Pessina F., Cabrini M., Wang Y., Capozzo I., Iannelli F. (2017). Damage-induced lncRNAs control the DNA damage response through interaction with DDRNAs at individual double-strand breaks. Nat. Cell Biol..

[143] Michalik K.M., Böttcher R., Förstemann K. (2012). A small RNA response at DNA ends in Drosophila. Nucleic Acids Res..

[144] D’Alessandro G., Whelan D.R., Howard S.M., Vitelli V., Renaudin X., Adamowicz M., Iannelli F., Jones-Weinert C.W., Lee M., Matti V. (2018). BRCA2 controls DNA:RNA hybrid level at DSBs by mediating RNase H2 recruitment. Nat. Commun..

[145] Ohle C., Tesorero R., Schermann G., Dobrev N., Sinning I., Fischer T. (2016). Transient RNA-DNA Hybrids Are Required for Efficient Double-Strand Break Repair. Cell.

[146] Francia S., Michelini F., Saxena A., Tang D., de Hoon M., Anelli V., Mione M., Carninci P., d’Adda di Fagagna F. (2012). Site-specific DICER and DROSHA RNA products control the DNA-damage response. Nature.

[147] Bader A.S., Bushell M. (2020). DNA:RNA hybrids form at DNA double-strand breaks in transcriptionally active loci. Cell Death Dis..

[148] Trott D.A., Porter A.C.G. (2006). Hypothesis: Transcript-templated repair of DNA double-strand breaks. BioEssays.

[149] Lavigne M.D., Konstantopoulos D., Ntakou-Zamplara K.Z., Liakos A., Fousteri M. (2017). Global unleashing of transcription elongation waves in response to genotoxic stress restricts somatic mutation rate. Nat. Commun..

[150] Liu X., Bushnell D.A., Kornberg R.D. (2013). RNA polymerase II transcription: Structure and mechanism. Biochim. Biophys. Acta.

[151] Becherel O.J., Yeo A.J., Stellati A., Heng E.Y., Luff J., Suraweera A.M., Woods R., Fleming J., Carrie D., McKinney K. (2013). Senataxin plays an essential role with DNA damage response proteins in meiotic recombination and gene silencing. PLoS Genet..

[152] Beli P., Lukashchuk N., Wagner S.A., Weinert B.T., Olsen J.V., Baskcomb L., Mann M., Jackson S.P., Choudhary C. (2012). Proteomic investigations reveal a role for RNA processing factor THRAP3 in the DNA damage response. Mol. Cell.

[153] Jain A., Bacolla A., Del Mundo I.M., Zhao J., Wang G., Vasquez K.M. (2013). DHX9 helicase is involved in preventing genomic instability induced by alternatively structured DNA in human cells. Nucleic Acids Res..

[154] Cohen S., Puget N., Lin Y.-L., Clouaire T., Aguirrebengoa M., Rocher V., Pasero P., Canitrot Y., Legube G. (2018). Senataxin resolves RNA:DNA hybrids forming at DNA double-strand breaks to prevent translocations. Nat. Commun..

[155] Lu W.-T., Hawley B.R., Skalka G.L., Baldock R.A., Smith E.M., Bader A.S., Malewicz M., Watts F.Z., Wilczynska A., Bushell M. (2018). Drosha drives the formation of DNA:RNA hybrids around DNA break sites to facilitate DNA repair. Nat. Commun..

[156] Rawal C.C., Zardoni L., Di Terlizzi M., Galati E., Brambati A., Lazzaro F., Liberi G., Pellicioli A. (2020). Senataxin Ortholog Sen1 Limits DNA:RNA Hybrid Accumulation at DNA Double-Strand Breaks to Control End Resection and Repair Fidelity. Cell Rep..

[157] Hegazy Y.A., Fernando C.M., Tran E.J. (2020). The balancing act of R-loop biology: The good, the bad, and the ugly. J. Biol. Chem..

[158] Meers C., Keskin H., Banyai G., Mazina O., Yang T., Gombolay A.L., Mukherjee K., Kaparos E.I., Newnam G., Mazin A. (2020). Genetic Characterization of Three Distinct Mechanisms Supporting RNA-Driven DNA Repair and Modification Reveals Major Role of DNA Polymerase ζ. Mol. Cell.

[159] Mazina O.M., Somarowthu S., Kadyrova L.Y., Baranovskiy A.G., Tahirov T.H., Kadyrov F.A., Mazin A.V. (2020). Replication protein A binds RNA and promotes R-loop formation. J. Biol. Chem..

[160] Keskin H., Meers C., Storici F. (2016). Transcript RNA supports precise repair of its own DNA gene. RNA Biol..

[161] Chandramouly G., Zhao J., McDevitt S., Rusanov T., Hoang T., Borisonnik N., Treddinick T., Lopezcolorado F.W., Kent T., Siddique L.A. (2021). Polθ reverse transcribes RNA and promotes RNA-templated DNA repair. Sci. Adv..

[162] Ferenczi A., Pyott D.E., Xipnitou A., Molnar A. (2017). Efficient targeted DNA editing and replacement in *Chlamydomonas reinhardtii* using Cpf1 ribonucleoproteins and single-stranded DNA. Proc. Natl. Acad. Sci. USA.

[163] Yang Y.G., Lindahl T., Barnes D.E. (2007). Trex1 exonuclease degrades ssDNA to prevent chronic checkpoint activation and autoimmune disease. Cell.

[164] Lanza D.G., Gaspero A., Lorenzo I., Liao L., Zheng P., Wang Y., Deng Y., Cheng C., Zhang C., Seavitt J.R. (2018). Comparative analysis of single-stranded DNA donors to generate conditional null mouse alleles. BMC Biol..

[165] Yang Y.G., Qi Y. (2015). RNA-directed repair of DNA double-strand breaks. DNA Repair.

[166] Shen Y., Nandi P., Taylor M.B., Stuckey S., Bhadsavle H.P., Weiss B., Storici F. (2011). RNA-driven genetic changes in bacteria and in human cells. Mutat. Res. Fundam. Mol. Mech. Mutagenesis.

[167] Mirgayazova R., Khadiullina R., Chasov V., Mingaleeva R., Miftakhova R., Rizvanov A., Bulatov E. (2020). Therapeutic Editing of the TP53 Gene: Is CRISPR/Cas9 an Option?. Genes.

[168] Brinkman E.K., Chen T., de Haas M., Holland H.A., Akhtar W., van Steensel B. (2018). Kinetics and Fidelity of the Repair of Cas9-Induced Double-Strand DNA Breaks. Mol. Cell.

[169] Zhu L., Mon H., Xu J., Lee J.M., Kusakabe T. (2015). CRISPR/Cas9-mediated knockout of factors in non-homologous end joining pathway enhances gene targeting in silkworm cells. Sci. Rep..

[170] Kostyushev D., Kostyusheva A., Brezgin S., Zarifyan D., Utkina A., Goptar I., Chulanov V. (2019). Suppressing the NHEJ pathway by DNA-PKcs inhibitor NU7026 prevents degradation of HBV cccDNA cleaved by CRISPR/Cas9. Sci. Rep..

[171] Li G., Liu D., Zhang X., Quan R., Zhong C., Mo J., Huang Y., Wang H., Ruan X., Xu Z. (2018). Suppressing Ku70/Ku80 expression elevates homology-directed repair efficiency in primary fibroblasts. Int. J. Biochem. Cell Biol..

[172] Maruyama T., Dougan S.K., Truttmann M.C., Bilate A.M., Ingram J.R., Ploegh H.L. (2015). Increasing the efficiency of precise genome editing with CRISPR-Cas9 by inhibition of nonhomologous end joining. Nat. Biotechnol..

[173] Riesenberg S., Chintalapati M., Macak D., Kanis P., Maricic T., Pääbo S. (2019). Simultaneous precise editing of multiple genes in human cells. Nucleic Acids Res..

[174] Charpentier M., Khedher A.H.Y., Menoret S., Brion A., Lamribet K., Dardillac E., Boix C., Perrouault L., Tesson L., Geny S. (2018). CtIP fusion to Cas9 enhances transgene integration by homology-dependent repair. Nat. Commun..

[175] Shao S., Ren C., Liu Z., Bai Y., Chen Z., Wei Z., Wang X., Zhang Z., Xu K. (2017). Enhancing CRISPR/Cas9-mediated homology-directed repair in mammalian cells by expressing Saccharomyces cerevisiae Rad52. Int. J. Biochem. Cell Biol..

[176] Tran N.-T., Bashir S., Li X., Rossius J., Chu V.T., Rajewsky K., Kühn R. (2019). Enhancement of Precise Gene Editing by the Association of Cas9 with Homologous Recombination Factors. Front. Genet..

[177] Savić N., Ringnalda F.C., Berk C., Bargsten K., Hall J., Jinek M., Schwank G. (2019). In vitro Generation of CRISPR-Cas9 Complexes with Covalently Bound Repair Templates for Genome Editing in Mammalian Cells. Bio-Protocol.

[178] Savic N., Ringnalda F.C.A.S., Lindsay H., Berk C., Bargsten K., Li Y., Neri D., Robinson M.D., Ciaudo C., Hall J. (2018). Covalent linkage of the DNA repair template to the CRISPR-Cas9 nuclease enhances homology-directed repair. eLife.

[179] Chen L., Chen J.-Y., Zhang X., Gu Y., Xiao R., Shao C., Tang P., Qian H., Luo D., Li H. (2017). R-ChIP Using Inactive RNase H Reveals Dynamic Coupling of R-loops with Transcriptional Pausing at Gene Promoters. Mol. Cell.

[180] Keskin H., Shen Y., Huang F., Patel M., Yang T., Ashley K., Mazin A.V., Storici F. (2014). Transcript-RNA-templated DNA recombination and repair. Nature.

[181] Butt H., Eid A., Ali Z., Atia M.A.M., Mokhtar M.M., Hassan N., Lee C.M., Bao G., Mahfouz M.M. (2017). Efficient CRISPR/Cas9-Mediated Genome Editing Using a Chimeric Single-Guide RNA Molecule. Front. Plant Sci..

[182] Li S., Li J., He Y., Xu M., Zhang J., Du W., Zhao Y., Xia L. (2019). Precise gene replacement in rice by RNA transcript-templated homologous recombination. Nat. Biotechnol..

[183] Daley J.M., Chiba T., Xue X., Niu H., Sung P. (2014). Multifaceted role of the Topo IIIα-RMI1-RMI2 complex and DNA2 in the BLM-dependent pathway of DNA break end resection. Nucleic Acids Res..

